# Bonding in Sisterhood: A Qualitative Study of a Virtual, Health-Related Program for Women of Color Amid COVID-19

**DOI:** 10.1093/geroni/igab046.1235

**Published:** 2021-12-17

**Authors:** Shanae Rhodes

**Affiliations:** UT Health San Antonio, San Antonio, Texas, United States

## Abstract

My Sister’s Keeper is an online education and support group created by women of color in response to disproportionate stresses related to COVID-19 experienced by women of color. The current study aims to examine the Stage 2 evidence that an online support group formed by members of the community may help mediate inequity-related stressors and increase receptiveness to health-related recommendations. To begin to develop this evidence, a thematic analysis of 8 in-depth individual interviews was performed. Resulting themes included: 1) feeling empowered; 2) solidarity in sisterhood (e.g., shared ownership of a virtual community); 3) being focused (on women of color) yet being inclusive; 4) currency of knowledge (e.g., responsibility to share knowledge with others); and 5) preferring virtual accessibility to stay connected. Preliminary data suggest that social support offered through an online platform dedicated to women of color can promote health during the pandemic and possibly beyond.

